# Rickettsiales in Ticks Removed from Outdoor Workers, Southwest Georgia and Northwest Florida, USA

**DOI:** 10.3201/eid2505.180438

**Published:** 2019-05

**Authors:** Elizabeth R. Gleim, L. Mike Conner, Galina E. Zemtsova, Michael L. Levin, Pamela Wong, Madeleine A. Pfaff, Michael J. Yabsley

**Affiliations:** Joseph W. Jones Ecological Research Center at Ichauway, Newton, Georgia, USA (E.R. Gleim, L.M. Conner);; Oxford College of Emory University, Oxford, Georgia, USA (E.R. Gleim, P. Wong);; University of Georgia, Athens, Georgia, USA (E.R. Gleim, M.A. Pfaff, M.J. Yabsley);; Centers for Disease Control and Prevention, Atlanta, Georgia, USA (G.E. Zemtsova, M.L. Levin)

**Keywords:** ticks, tick bites, tickborne infections, vectorborne infections, outdoor workers, Rickettsiales, Rickettsia, Ehrlichia, zoonoses, Georgia, Florida, United States

## Abstract

We determined the prevalence of selected Rickettsiales in 362 ticks removed from outdoor workers in southwest Georgia and northwest Florida, USA. Persons submitted an average of 1.1 ticks/month. We found *Ehrlichia chaffeensis* in an *Amblyomma maculatum* tick, and Panola Mountain *Ehrlichia* sp. in 2 *A. maculatum* ticks and 1 *Dermacentor variabilis* tick.

The southeastern United States has multiple tick species that can transmit pathogens to humans. The most common tick species, *Amblyomma americanum*, is the vector for the causative agents of human ehrlichioses and southern tick-associated rash illness, among others (*1*). *Dermacentor variabilis* ticks can transmit the causative agent of Rocky Mountain spotted fever, and *Ixodes scapularis* ticks can transmit the causative agents of Lyme disease, babesiosis, and human granulocytic anaplasmosis (*1*). Although less common in the region, *A. maculatum* ticks are dominant in specific habitats and can transmit the causative agent of *Rickettsia parkeri* rickettsiosis (*1*).

Persons who have occupations that require them to be outside on a regular basis might have a greater risk for acquiring a tickborne disease (*2*). Although numerous studies have been conducted regarding risks for tickborne diseases among forestry workers in Europe, few studies have been performed in the United States (*2*,*3*). The studies that have been conducted in the United States have focused on forestry workers in the northeastern region (*2*). However, because of variable phenology and densities of ticks, it is useful to evaluate tick activity and pathogen prevalence in various regions and ecosystems.

Burn-tolerant and burn-dependent ecosystems, such as pine (*Pinus* spp.) and mixed pine forests commonly found in the southeastern United States, have unique tick dynamics compared with those of other habitats (*4*). The objective of this study was to determine the tick bite risk and tickborne pathogen prevalence in ticks removed from forestry workers working in pine and mixed pine forests in southwest Georgia and northwest Florida, USA.

During June 2009–December 2011, forestry workers in southwestern Georgia (7 counties) and northwestern Florida (1 county) submitted ticks crawling on or attached to them. We identified ticks and tested them for selected pathogens (Appendix). Immature forms of the same species from the same day and person were pooled (<5 nymphs and <20 larvae) for testing.

A total of 53 persons submitted 362 ticks (Table). Excluding larvae, the most common tick species submitted was *A. maculatum*, followed by *A. americanum*, *I. scapularis*, and *D. variabilis*. On 4 occasions, 1 person submitted *A. tuberculatum* ticks (3 batches of larvae and 1 batch of nymphs) from a longleaf pine site in Baker County, Georgia. Average submissions per persons were 2.6 ticks (median 1 tick), but 1 person submitted 100 ticks. A total of 24 persons submitted ticks more than once, and they submitted an average of 0.08–6.5 ticks/month (overall average submission rate of 1.1 ticks/month). Three ticks were engorged (1 *D. variabilis* adult, 1 *A. americanum* nymph, and 1 *Amblyomma* sp. nymph); only the *Amblyomma* sp. nymph was positive for a pathogen (*R. amblyommatis*).

**Table:** Prevalence of *Ehrlichia chaffeensis,* PME, and *Rickettsia* spp. in ticks submitted by outdoor workers, southwestern Georgia and northwestern Florida, USA*

Tick species and stage	Months submitted	No. positive ticks/no. tested (%)			*Rickettsia* spp.†
		*E. chaffeensis*	PME	*Rickettsia* spp.	
*Amblyomma americanum*, adults	Feb–Sep	0/11 (0)	0/11 (0)	4/11 (36.4)	2 *R. amblyommatis*
*A. americanum* nymphs‡	Mar–Sep	0/43 (0)	0/43 (0)	12/43 (27.9)	9 *R. amblyommatis*
*A. americanum* larvae‡	Apr and Oct	0/5 (0)	0/5 (0)	1/5 (20.0)	1 *R.amblyommatis*
*Amblyomma* sp*.* nymphs	Jun and Oct	0/3 (0)	0/3 (0)	1/3 (33.3)	1 *R. amblyommatis*§
*Amblyomma* sp*.* larvae	Oct	0/5 (0)	0/5 (0)	0/5 (0)	
*A. maculatum* adults	May–Oct	1/83 (1.2)	2/83 (2.4)¶	18/83 (21.7)	5 *R. amblyommatis*,§ 4 *R. parkeri*,§ 1 *Rickettsia* sp. TR-39/TX125, 2 *Candidatus* R. andeanae
*A. tuberculatum* nymphs‡	Apr	0/5 (0)	0/5 (0)	1/5 (5.0)	1 novel SFG *Rickettsia* sp.
*A. tuberculatum* larvae‡	Feb#	0/182 (0)	0/182 (0)	10/182 (5.5)**	10 novel SFG *Rickettsia* sp.**
*Dermacentor variabilis* adults	Jun–Aug	0/10 (0)	1/10 (10.0)	2/10 (20.0)	1 *R. amblyommatis*§
*Ixodes scapularis* adults	Oct–Mar	NT	0/15 (0)	7/15 (46.7)	4 *Rickettsia* sp. TR-39, 3 *R. buchneri*

*All *Rickettsia* spp. were identified by sequencing unless otherwise noted. NT, not tested; PME, Panola Mountain *Ehrlichia* sp., SFG, spotted fever group. †*Rickettsia* spp. for whom amplicons did not provide high-quality bidirectional sequences were categorized as unknown *Rickettsia* spp. ‡Minimum infection prevalence is no. positive tick pools/no. ticks tested. §The following *R. amblyommatis* samples were identified by restriction fragment length polymorphism analysis: for 1 *D. variabilis* adult, 5 *A. maculatum* adults, and 1 *Amblyomma* sp. nymph; for *A. americanum*, 1 adult, 2 nymphs, and 1 larva. Three *A. maculatum* adults were also identified as containing *R. parkeri* positive by restriction fragment length polymorphism analysis. ¶Data included in Loftis et al. (*5*). #Date was known only for 1 submission of 20 larvae. Dates for others were not provided when submitted. **Data included in Zemtsova et al. (*6*).

*Rickettsia* spp. prevalence was 36.4% in adult, 27.9% in nymphal, and 20% in larval *A. americanum* ticks; *R. amblyommatis* was the only species identified (Table). *Rickettsia* spp. were detected in 23% of *A. maculatum* adults; *R. amblyommatis* was most common (6.0%), followed by *R. parkeri* (4.8%). A previously detected novel *Rickettsia* sp. was identified in 10 of 11 *A. tuberculatum* larval pools and was reported by Zemtsova et al. (*6*). An additional pool of *A. tuberculatum* nymphs was tested in this study and also was positive for the novel *Rickettsia* sp. *E. chaffeensis* was detected in 1 *A. maculatum* adult (prevalence 1.2%), and Panola mountain *Ehrlichia* sp. was detected in 2 *A. maculatum* adults (prevalence 2.4%) and 1 *D. variabilis* adult (prevalence 10%). No ticks were positive for *Borrelia* spp., *E. ewingii*, or *Anaplasma phagocytophilum*.

Thus, forestry workers were found to encounter ticks on a regular basis, and peak encounter rates reflected previously reported tick seasonality in this region (*4*). Only 3 (0.8%) of the ticks submitted were engorged, indicating prompt removal of most ticks and thus low risk for pathogen transmission. *A. maculatum*, a fairly uncommon tick in the southeastern United States, was the most commonly submitted tick. However, *A. maculatum* ticks dominate in regularly burned pine ecosystems (*4*), which is where most of these workers spent their time.

We observed several unique findings related to pathogens during this study. Larvae and nymphs of *A. tuberculatum* ticks were submitted on multiple occasions, a tick rarely reported on humans (*7*). These findings in conjunction with the identification of a novel *Rickettsia* sp. (*6*), suggest that additional research is warranted. This study also identified *E. chaffeensis* and Panola Mountain *Ehrlichia* in *A. maculatum* ticks. Although *A. americanum* ticks are considered the primary vector of *Ehrlichia* spp., these pathogens have been occasionally reported in questing *A. maculatum* ticks, suggesting that this tick might be involved in their transmission cycles (*5*,*8*). We also detected Panola Mountain *Ehrlichia* in 1 *D. variabilis* tick. Thus, further research regarding these alternative tick species as potential vectors of these pathogens is warranted, particularly in the case of *A. maculatum* ticks, which were a common species on forestry workers and are widespread in this region (*4*).

Appendix Additional information on Rickettsiales in ticks removed from outdoor workers, southwest Georgia and northwest Florida, USA.
