# Supplementary material for: Rickettsiales in Ticks Removed from Outdoor Workers, Southwest Georgia and Northwest Florida, USA
Source: Emerg Infect Dis. 2019 May;25(5):1019–21. doi: 10.3201/eid2505.180438 (PMC6478216; doi:10.3201/eid2505.180438)
Supplement: Appendix — Additional information on Rickettsiales in ticks removed from outdoor workers, southwest Georgia and northwest Florida, USA. [file 18-0438-Techapp-s1.pdf]

Article DOI: https://doi.org/10.3201/eid2505.180438

Rickettsiales in Ticks Removed from Outdoor Workers, Southwest Georgia and Northwest Florida, USA

Technical Appendix

Sample Acquisition

For tick collections, submissions were accepted from forestry and wildlife workers who worked throughout the region (Baker, Calhoun, Stewart, Thomas, Decatur, Dooly, Macon Counties in Georgia and Gladsden County in Florida). We notified participants about the project by emails and meetings. Workers were given tubes of 70% ethanol and asked to store and submit ticks in these tubes if they wished to participate in the project. Workers were instructed to separate ticks by date and location (if possible). The date(s) and location(s) where workers had obtained the tick(s) were recorded. No other information (e.g., time spent outdoors, past information on tick infestations or tick bites, etc.) was provided.

Tick Identification and Pathogen Testing

We morphologically identified nonlarval *Dermacentor variabilis* and *Amblyomma* spp. ticks. *Amblyomma* larvae and *Ixodes* spp. (all stages) were identified by using PCR targeting the 16S rRNA gene and sequencing (*1*). Ticks submitted in 2009 and 2010 were tested for *Rickettsia* spp., *Ehrlichia chaffeensis*, and *E. ewingii* by using a multiplex quantitative PCR targeting the 17-kDa gene of *Rickettsia* spp. and the 16S rRNA gene of *Ehrlichia* spp. (*2*).

Samples positive for *Rickettsia* spp. were analyzed by using a restriction fragment length polymorphism assay targeting the major outer membrane protein A gene by using primers RR190.70 and RR190.701R (*3*), followed by digestion with restriction enzymes *RsaI* and *Pst*I (*4*). Ticks collected in 2011 were tested for *Rickettsia* spp., *Ehrlichia chaffeensis*, and *E. ewingii* by using nested PCRd specific for the 17-kDa (*Rickettsia* spp.) or 16S rRNA genes (*Ehrlichia* spp.) (*4*). Any samples positive for *Rickettsia* spp. that were not successfully identified by 17-kDa sequencing were then tested by using PCR and sequencing of the outer membrane protein A

gene (*5*). For all ticks, the *gltA* gene of Panola Mountain *Ehrlichia* sp., the *fla* gene of *Borrelia* spp., and the *msp2* gene of *Anaplasma phagocytophilum* were targeted (*1*).

References

1. Yabsley MJ, Nims TN, Savage MY, Durden LA. Ticks and tick-borne pathogens and putative symbionts of black bears (*Ursus americanus floridanus*) from Georgia and Florida. J Parasitol. 2009;95:1125–8. PubMed http://dx.doi.org/10.1645/GE-2111.1

2. Killmaster LF, Loftis AD, Zemtsova GE, Levin ML. Detection of bacterial agents in *Amblyomma americanum* (Acari: Ixodidae) from Georgia, USA, and the use of a multiplex assay to differentiate *Ehrlichia chaffeensis* and *Ehrlichia ewingii*. J Med Entomol. 2014;51:868–72. PubMed http://dx.doi.org/10.1603/ME13225

3. Roux V, Fournier PE, Raoult D. Differentiation of spotted fever group rickettsiae by sequencing and analysis of restriction fragment length polymorphism of PCR-amplified DNA of the gene encoding the protein rOmpA. J Clin Microbiol. 1996;34:2058–65. PubMed

4. Eremeeva M, Yu X, Raoult D. Differentiation among spotted fever group rickettsiae species by analysis of restriction fragment length polymorphism of PCR-amplified DNA. J Clin Microbiol. 1994;32:803–10. PubMed

5. Regnery RL, Spruill CL, Plikaytis BD. Genotypic identification of rickettsiae and estimation of intraspecies sequence divergence for portions of two rickettsial genes. J Bacteriol. 1991;173:1576–89. PubMed http://dx.doi.org/10.1128/jb.173.5.1576-1589.1991
